# Ingestion of probiotic (*Lactobacillus helveticus* and *Bifidobacterium longum*) alters intestinal microbial structure and behavioral expression following social defeat stress

**DOI:** 10.1038/s41598-021-83284-z

**Published:** 2021-02-12

**Authors:** Katherine A. Partrick, Anna M. Rosenhauer, Jérémie Auger, Amanda R. Arnold, Nicole M. Ronczkowski, Lanaya M. Jackson, Magen N. Lord, Sara M. Abdulla, Benoit Chassaing, Kim L. Huhman

**Affiliations:** 1 Neuroscience Institute, Center for Behavioral Neuroscience, Georgia State University, PO Box 5030, Atlanta, GA 30303-5030 USA; 2 Rosell Institute for Microbiome and Probiotics, Montreal, QC Canada; 3 INSERM U1016, Team “Mucosal Microbiota in Chronic Inflammatory Diseases”, CNRS UMR 8104, Université de Paris, Paris, France; 4 Institute for Biomedical Sciences, Georgia State University, Atlanta, GA USA

**Keywords:** Microbiology, Neuroscience

## Abstract

Social stress exacerbates anxious and depressive behaviors in humans. Similarly, anxiety- and depressive-like behaviors are triggered by social stress in a variety of non-human animals. Here, we tested whether oral administration of the putative anxiolytic probiotic strains *Lactobacillus helveticus* R0052 and *Bifidobacterium longum* R0175 reduces the striking increase in anxiety-like behavior and changes in gut microbiota observed following social defeat stress in Syrian hamsters. We administered the probiotic at two different doses for 21 days, and 16S rRNA gene amplicon sequencing revealed a shift in microbial structure following probiotic administration at both doses, independently of stress. Probiotic administration at either dose increased anti-inflammatory cytokines IL-4, IL-5, and IL-10 compared to placebo. Surprisingly, probiotic administration at the low dose, equivalent to the one used in humans, significantly *increased* social avoidance and decreased social interaction. This behavioral change was associated with a reduction in microbial richness in this group. Together, these results demonstrate that probiotic administration alters gut microbial composition and may promote an anti-inflammatory profile but that these changes may not promote reductions in behavioral responses to social stress.

## Introduction

The human gastrointestinal tract houses a vastly abundant community of microorganisms, and it has become increasingly clear that the state of this microbial community can meaningfully impact disease states^1–3^. This community of microbes is necessary for general health and vital processes such as digestion^4,5^, gastrointestinal barrier protection, and immunoregulation^6,7^. While it is also known that multiple, bidirectional routes of communication exist between the gastrointestinal tract, its microbiota, and the brain^8^, more research is required to understand the neurological and behavioral consequences of this communication. More recent data demonstrate that the gut microbiota may alter brain and behavior^9–12^, but few studies have examined whether this community modifies social behavior in ethologically relevant models of social interaction.

Social stress is the primary form of stress experienced by humans and is a major predictor for the onset of a variety of neuropsychiatric disorders^13–15^, such as mood and anxiety disorders. Animal models of social stress have revealed that socially defeated rodents and monkeys display behavioral responses (i.e., social avoidance, changes in ingestive behavior and sleep, and increases in anxiety- and depressive-like behaviors) that resemble the symptoms of neuropsychiatric disorders in humans^15–21^. Extensive research has explored how social stress may increase the likelihood of developing a mood or anxiety disorder^15,22,23^; however, data on the direct effect of gut microbiota on susceptibility to social stress are limited. This deficiency is important because it has been suggested that the gut microbial community could be a potential target for the development of novel treatments (e.g., “psychobiotics”) for these neuropsychiatric disorders^24,25^. It is known that the gut is responsive to stress and that stress-induced dysbiosis of the gut microbial community is linked to negative health consequences such as breakdown of the gastrointestinal barrier and a heightened proinflammatory profile^26–31^. Our lab recently demonstrated that even a single social defeat in Syrian hamsters causes dysbiosis of the gut microbial community in both animals that win (e.g., become dominant) and those that lose (e.g., become subordinate), with such alterations being exacerbated following repeated bouts of social stress^32^. Importantly, specific microbial taxa also appear to predict future dominant or subordinate status following an agonistic encounter, suggesting that the microbial profile may modify future behavioral responses to social conflict^32^.

In order to better understand how the state of the gut microbial community drives social behavior and to assess whether “psychobiotics” might protect against the deleterious effects of social stress, we asked whether manipulating the gut microbial community with a probiotic containing large quantities of gut-derived microbes that are thought to benefit the host^33^ would promote resistance to the behavioral consequences of social stress. We used a well-characterized social defeat model in Syrian hamsters^16,32,34,35^. This species provides an ideal model of social stress because when conspecifics are paired, they readily produce aggressive and territorial behavior that rapidly results in the formation of a stable dominance relationship^36^. Importantly, agonistic behavior during these brief encounters is highly ritualized and rarely results in tissue damage, allowing us to focus on the psychological, as opposed to physical, aspects of social stress. This advantage also eliminates any confounding effect of physical injury on inflammation and the gut microbial community. Here, we tested whether oral treatment with a probiotic containing *Lactobacillus helveticus* R0052 and *Bifidobacterium longum* R0175 is sufficient to reduce stress-induced dysbiosis of the gut microbial community and to decrease defeat-induced social avoidance. Furthermore, we investigated the extent to which probiotic treatment impacts the gut microbial community or has an anti-inflammatory effect.

## Results

### Probiotic intervention at a low dose increases susceptibility to social stress

**Figure 1:**
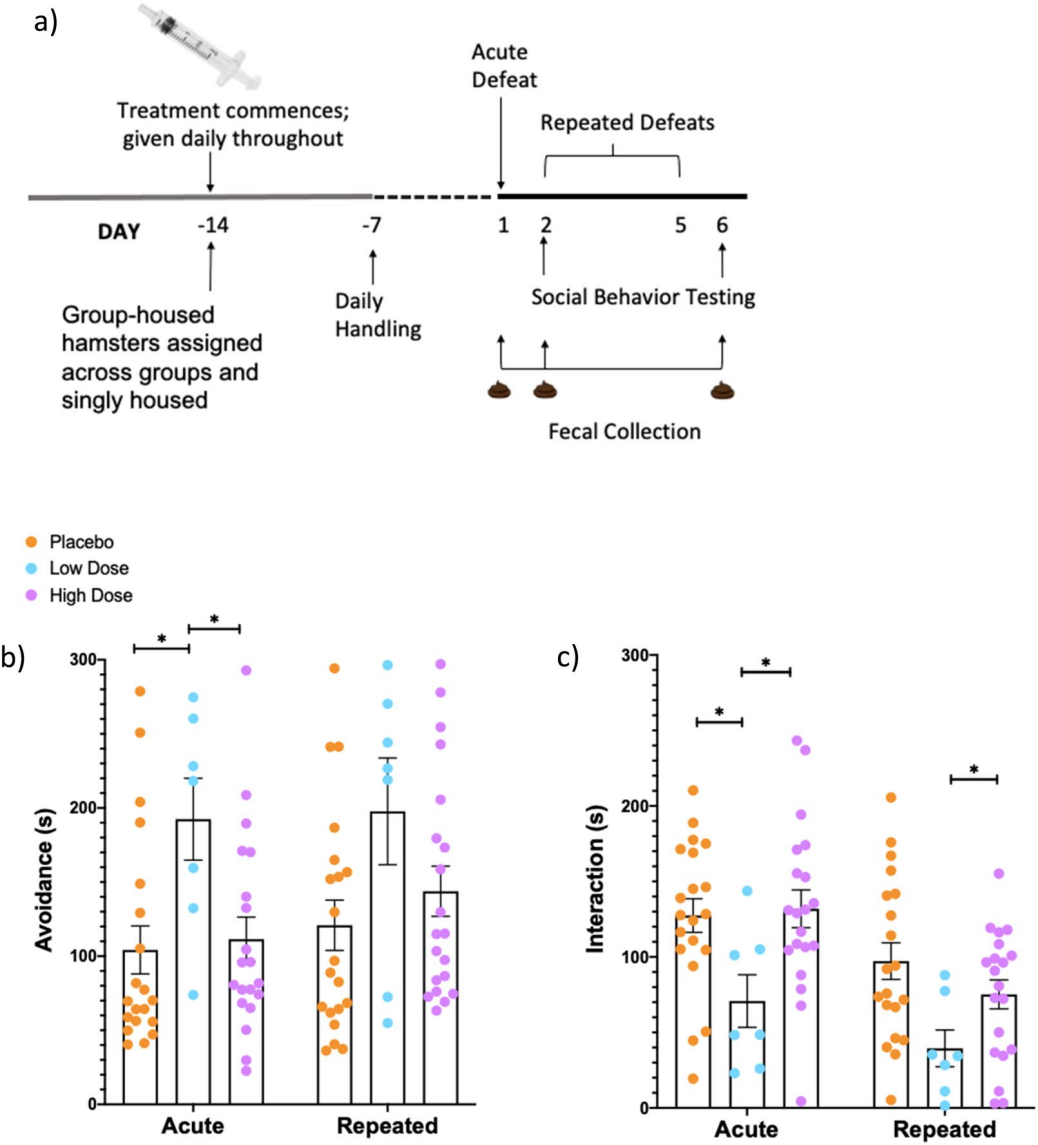
Probiotic intervention at a low dose increases susceptibility to social stress. (**a**) A timeline for the study is shown in (**a**). Following the acute defeat, hamsters treated with a low dose of the probiotic (*n* = 7) (blue dots) avoided a novel opponent more (**b**) and interacted with the opponent less (**c**) than did hamsters treated with a high dose of the probiotic (*n* = 20) (purple dots) or the placebo (*n* = 20) (orange dots) (*p < 0.05). Following repeated defeats, there were no significant differences in avoidance behavior between treatment groups (**b**), yet hamsters treated with the lose dose (n = 7) (blue dots) interacted with a novel opponent significantly less than did placebo-treated hamsters (*n* = 20) (orange dots) (**c**; *p < 0.05). Data presented as mean +/− standard error of the mean. Note that the videos for three animals in the low dose probiotic group were lost due to a technical problem, so the group n for behavioral analysis is lower than for the other measures analyzed.

A timeline for the study is shown in Fig. 1a. Following both acute and repeated defeat training, hamsters were tested for social avoidance and social interaction with a novel, caged opponent. Repeated Measures, Two-way ANOVAs with Tukey’s post hoc analyses were run to assess the effect of probiotic intervention (high and low dose or placebo) and number of defeats (acute or repeated) on social behavior. Behavioral data from 3 hamsters in the low dose probiotic group were not scored due to a technical error in saving the videos and thus the number of animals in this group was reduced. Analysis of social avoidance behavior revealed no interaction effect (*F*(2, 44) = 0.5, *p* = 0.6, η^2^ = 0.02) or main effect of defeat number (*F*(1, 44) = 2.4, *p* = 0.1, η^2^ = 0.05) on avoidance behavior; however, there was a significant main effect of probiotic treatment (*F*(2, 44) = 4.2, *p* = 0.02, η^2^ = 0.2). Tukey’s pairwise comparisons indicated that hamsters treated with the low dose of the probiotic exhibited significantly more social avoidance following an acute defeat than did hamsters treated with a high dose of the probiotic (*p* = 0.04) or the placebo (*p* = 0.02) (Fig. 1b). Following repeated defeats, no significant differences in avoidance behavior between treatment groups were observed; however, there was a strong trend for hamsters treated with the low dose of the probiotic to spend significantly more time avoiding a caged opponent compared to placebo-treated hamsters (*p* = 0.055) (Fig. 1b). Analysis of social interaction revealed no interaction effect (*F*(2, 44) = 1.5, *p* = 0.2, η^2^ = 0.06), but there was a significant main effect of defeat number (*F*(1, 44) = 22.3, *p* < 0.0001, η^2^ = 0.3) and a main effect of probiotic treatment on social interaction (*F*(2, 44) = 4.8, *p* = 0.01, η^2^ = 0.2). Tukey’s post hoc analysis revealed that hamsters treated with a low dose of the probiotic spent significantly less time interacting socially with a caged opponent following an acute defeat compared to hamsters treated with a high dose of the probiotic (*p* = 0.02) or placebo (*p* = 0.03) (Fig. 1b). Following repeated defeats, hamsters treated with a low dose of the probiotic exhibited less social interaction than did placebo-treated hamsters (*p* = 0.03), but they did not differ from hamsters receiving the high dose of the probiotic (p = 0.2) (Fig. 1c).

### Probiotic intervention modestly alters the concentration of probiotic strains in feces

**Figure 2:**
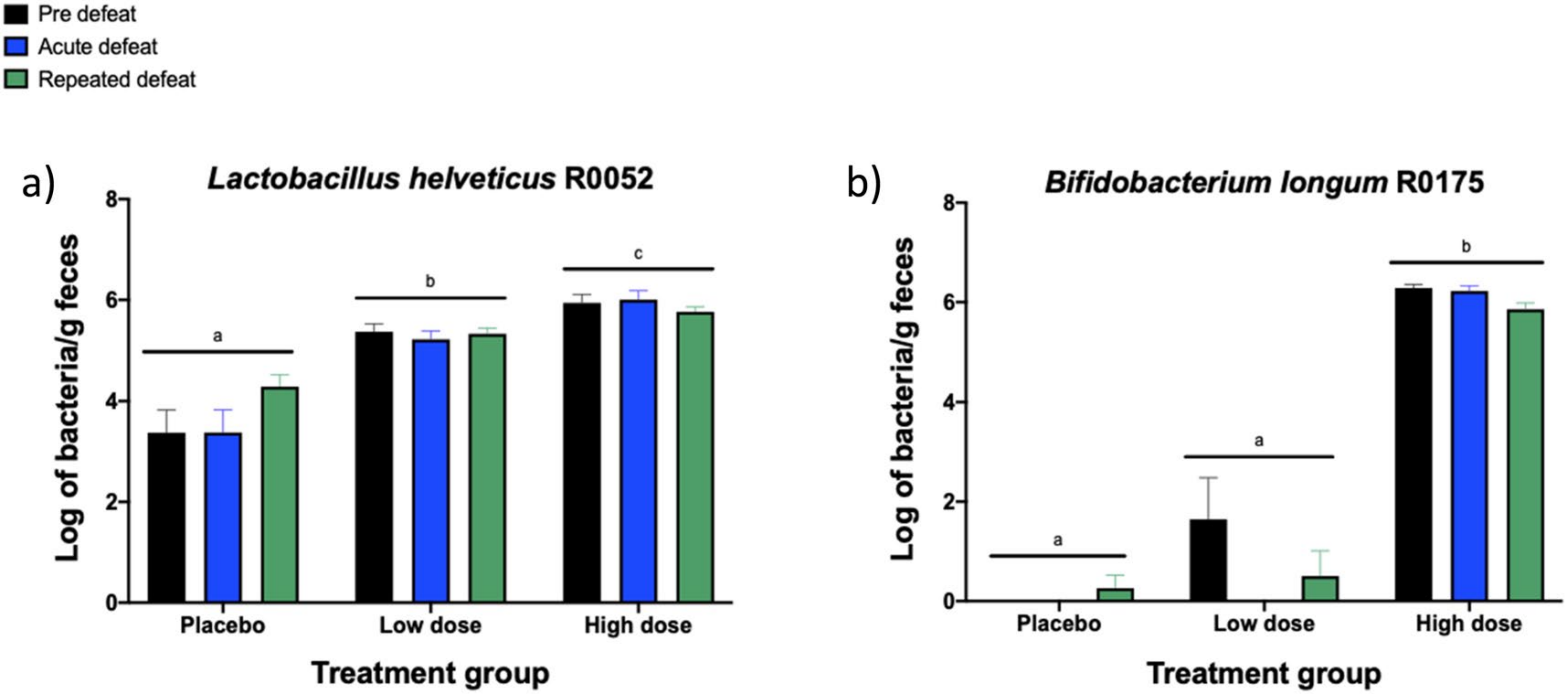
Probiotic intervention modestly alters the concentration of probiotic strains in feces. Concentrations of *Lactobacillus helveticus* R0052 (**a**) and *Bifidobacterium longum* R0175 (**b**) detected in fecal samples collected approximately 1 h before the acute defeat (pre defeat, black bar), 24 h following the acute defeat (blue bar), or 24 h following repeated defeats (green bar). Data are presented as mean +/− standard error of the mean; non-shared letters within each panel denote a significant difference (*p* < 0.03).

Measurement of the probiotic strains in fecal samples collected from each hamster approximately 1 h before the acute defeat, 24 h after the acute defeat, or 24 h after the final repeated defeat was completed by qPCR. Concentrations were not normally distributed. Therefore, nonparametric Kruskal–Wallis with Dunn’s multiple comparisons test was used to compare the concentrations of probiotic strains across treatment groups. Concentrations of the probiotic strains for each treatment group were collapsed across the three time points for statistical analysis. Hamsters whose concentrations were below the range of the standard curve were given a value of 0 for analysis. Concentrations of unspiked samples were 6.44 log bacteria/g feces for *Lactobacillus helveticus* R0052 and 6.49 log bacteria/g feces for *Bifidocaterium logum* R0175. A significant effect of treatment was observed for the concentration of *Lactobacillus helveticus* R0052 (*H*(3,150) = 102.9, *p* < 0.0001). The concentration of *Lactobacillus helveticus* R0052 was significantly lower in the placebo group compared to the two probiotic groups and the concentration of *Lactobacillus helveticus* R0052 was higher in hamsters treated with the high dose of the probiotic compared to hamsters treated with the low dose of the probiotic or placebo (Dunn’s multiple comparisons test; high dose v placebo, *p* < 0.0001, low dose v placebo, *p* < 0.0001, high dose v low dose, *p* = 0.028, Fig. 2a). For *Bifidobacterium longum* R0052, a significant effect of treatment was also observed (*H*(3,150) = 129.3, *p* < 0.0001); the concentration of *Bifidobacterium longum* R0052 was higher in the feces of hamsters treated with the high dose of the probiotic compared to the hamsters treated with the low dose of the probiotic or placebo (Dunn’s multiple comparisons test; high dose v placebo, *p* < 0.0001, high dose v low dose, *p* < 0.0001, Fig. 2b).

### Probiotic intervention alters gut microbiota composition

**Figure 3:**
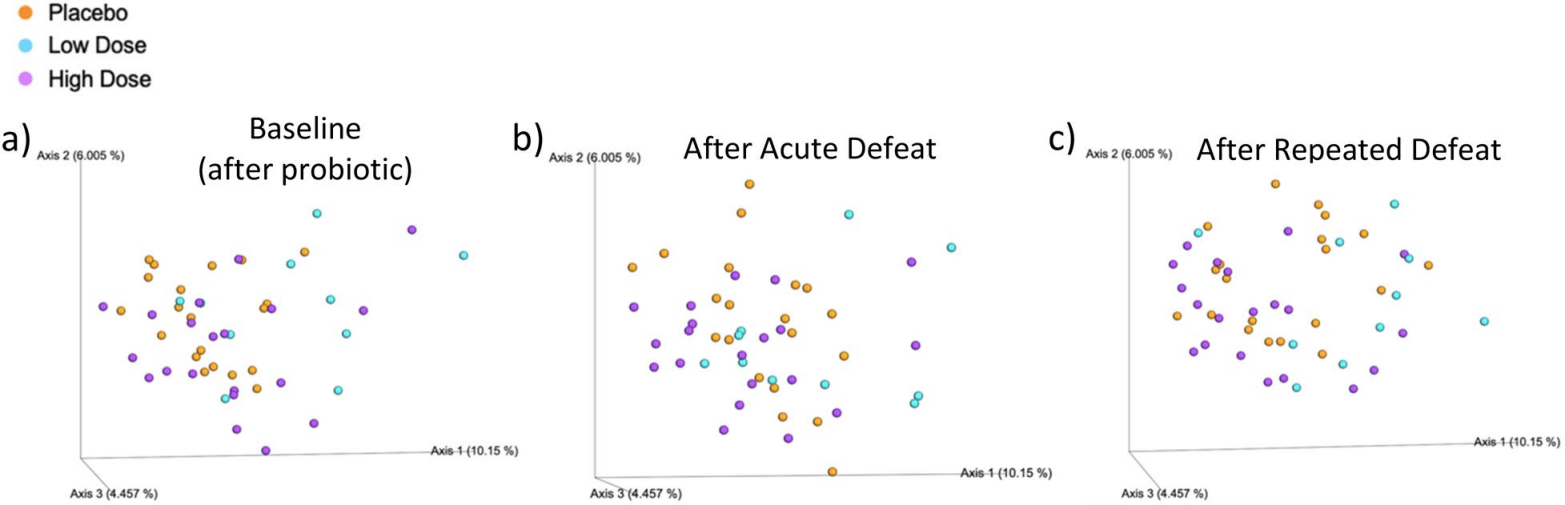
Probiotic intervention alters the gut microbial composition before and after social stress. Principle coordinate analysis (PCoA) of the unweighted UniFrac distance at baseline (**a**), after acute social stress (**b**), and after repeated social stress (**c**). Although not immediately obvious based on the PCoA plots, the unweighted UniFrac distance revealed that at “baseline” (after probiotic treatment but before any behavioral manipulations) the microbial composition of each treatment group differed from one another (**a**) and that, after acute social stress, the microbial composition of hamsters administered the low dose of the probiotic (*n* = 10) (orange dots) differed from that of hamsters administered the high dose (*n* = 20) (red dots) (**b**). Following repeated social stress, unweighted UniFrac revealed that the microbial composition of hamsters treated with a low dose of the probiotic (*n* = 10) (orange dots) was different than that of both the placebo-treated hamsters (*n* = 20) (blue dots) and the hamsters treated with a high dose of the probiotic (*n* = 20) (red dots) (**c**). *P* values were determined using PERMANOVA analysis and denoted as significant at *p* < 0.05 (**a**–**c**).

Microbiota composition was analyzed by 16S rRNA Illumina sequencing of fecal DNA samples collected before defeat training (baseline), after the initial (acute) defeat, and after nine (repeated) bouts of social defeat. PERMANOVA analysis of the unweighted UniFrac distance revealed that the microbial composition of all treatment groups significantly differed from one another after probiotic treatment but before any behavioral manipulation (low dose v high dose, *p* = 0.006; low dose v placebo, *p* = 0.006; high dose v placebo, *p* = 0.01) (Fig. 3a). Following acute defeat, the microbial composition of hamsters given the low dose of the probiotic differed significantly from that of hamsters administered the high dose (*p* = 0.05) (Fig. 3b). After the acute defeat there was also a strong trend for the microbial composition of hamsters given either dose of the probiotic to significantly differ from that of placebo-treated hamsters (low dose v placebo, *p* = 0.057; high dose v placebo, *p* = 0.057) (Fig. 3b). Following repeated defeats, the microbial composition of hamsters treated with a low dose of the probiotic differed from that of both hamsters treated with a high dose of the probiotic (*p* = 0.006) and placebo-treated hamsters (*p* = 0.04) (Fig. 3c).

**Figure 4:**
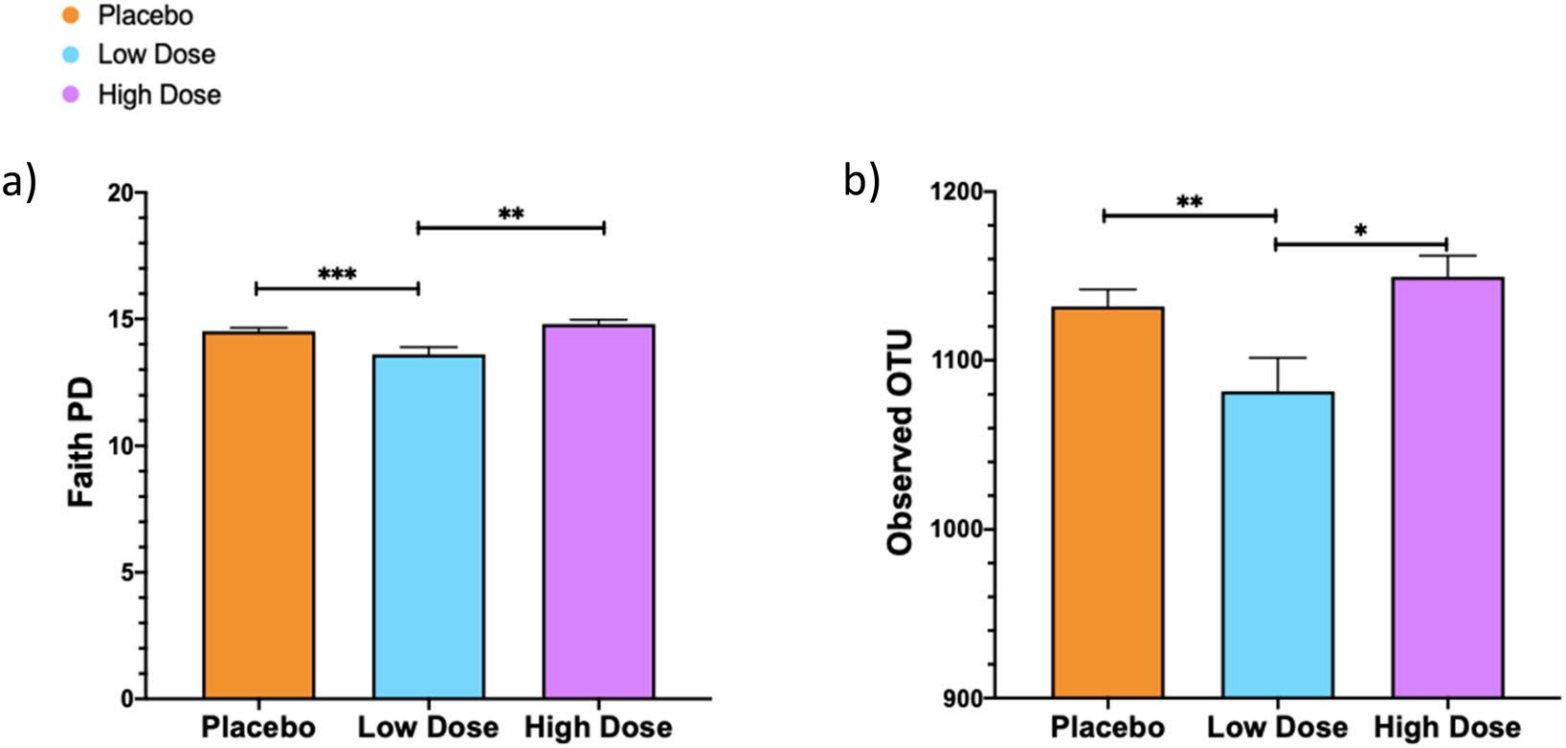
Probiotic administration at a low dose decreases intestinal microbiota diversity. Alpha diversity was determined by Faith PD Whole Tree and Observed OTUs in hamsters treated with a high dose (purple) or low dose (blue) of the probiotic and in placebo-treated hamsters (orange). No differences within groups were observed, thus groups were collapsed across time points. Both Faith PD (**a**) and Observed OTUs (**b**) indicated that hamsters given a low dose of the probiotic (blue) had lower diversity than did hamsters given a high dose (purple) or placebo (orange). *denotes *p* < 0.05; **denotes *p* ≤ 0.01; ***denotes *p* ≤ 0.001. Data presented as mean +/− standard error of the mean.

The analysis of alpha diversity of the intestinal microbiota, reflecting the bacterial richness and evenness of the community, revealed a significant effect of probiotic treatment using both phylogeny-based (Faith Phylogenetic Diversity (PD) Whole Tree) (*H* = 14.54, *p* = 0.0007) and non-phylogeny-based (Observed Operational Taxonomic Units (OTUs)) (*H* = 10.33, *p* = 0.006) measurements. Dunn’s multiple comparisons test revealed a significant decrease in alpha diversity for defeated hamsters treated with a low dose of the probiotic compared to defeated hamsters treated with a high dose of the probiotic (Faith PD Whole Tree, *p* = 0.001; Observed OTUs, *p* = 0.01) or with the placebo (Faith PD Whole Tree, *p* = 0.008; Observed OTUs, *p* = 0.02) (Fig. 4).

**Figure 5:**
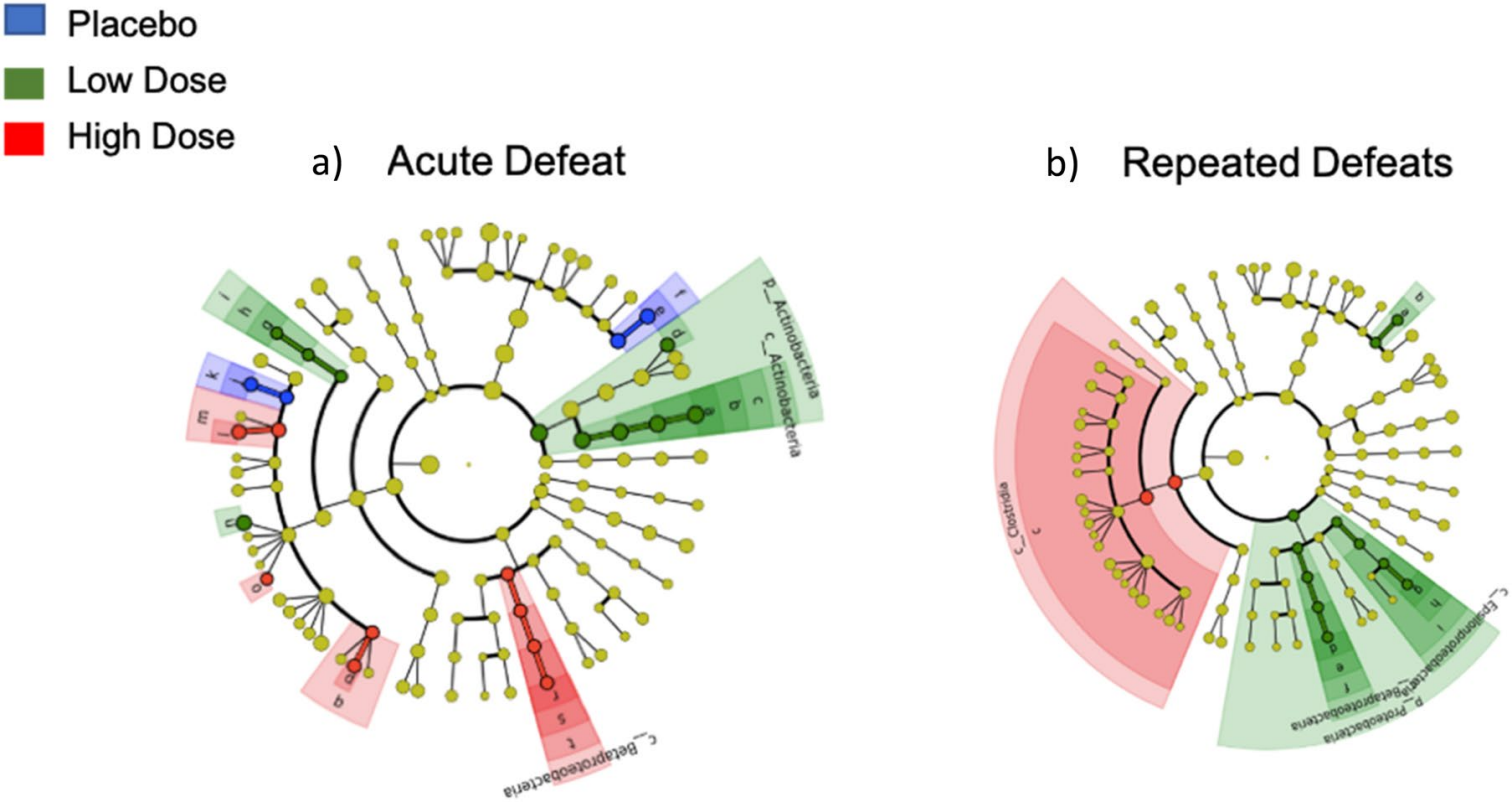
Identification of microbial taxa that were differentially altered across groups following acute or repeated social defeat. LEfSE analysis (Version 1, available at: https://huttenhower.sph.harvard.edu/galaxy/))^82^ was used to identify microbial taxa that differed among or between groups in hamsters given either a low dose of the probiotic (green), high dose of the probiotic (red), or placebo (blue) after an acute defeat (**a**) or repeated defeats (**b**). Blue represents taxa higher in placebo-treated hamsters compared to both doses of the probiotic; green, taxa higher in hamsters treated with a low dose of the probiotic compared to the high dose or placebo; red, taxa higher in hamsters treated with a high dose of the probiotic compared to the low dose or placebo. Only taxa meeting an LDA significant threshold of  > 2.0 are represented.

Next, LEfSE analysis (Linear Discriminant Analysis (LDA) Effect Size) was used to identify bacterial taxa that were significantly altered by treatment or defeat training. An LDA threshold of 2 was used to infer significance (LDA > 2, *p* < 0.05). A relatively small number of taxa were significantly altered by probiotic treatment using this stringent threshold. Notably, genus *Bifidobacterium* was significantly higher in hamsters treated with a low dose of the probiotic compared to all other treatment groups following acute defeat (LDA > 2, *p* < 0.05; Fig. 5a). Following repeated defeats, genus *Prevotella* was significantly higher in hamsters treated with a high dose of the probiotic compared to their pre-defeat baseline and phyla Proteobacteria was significantly higher in placebo-treated animals compared to both probiotic treatment groups (LDA > 2, *p* < 0.05; Fig. 5b).

### Probiotic intervention increases circulating anti-inflammatory cytokines

**Figure 6:**
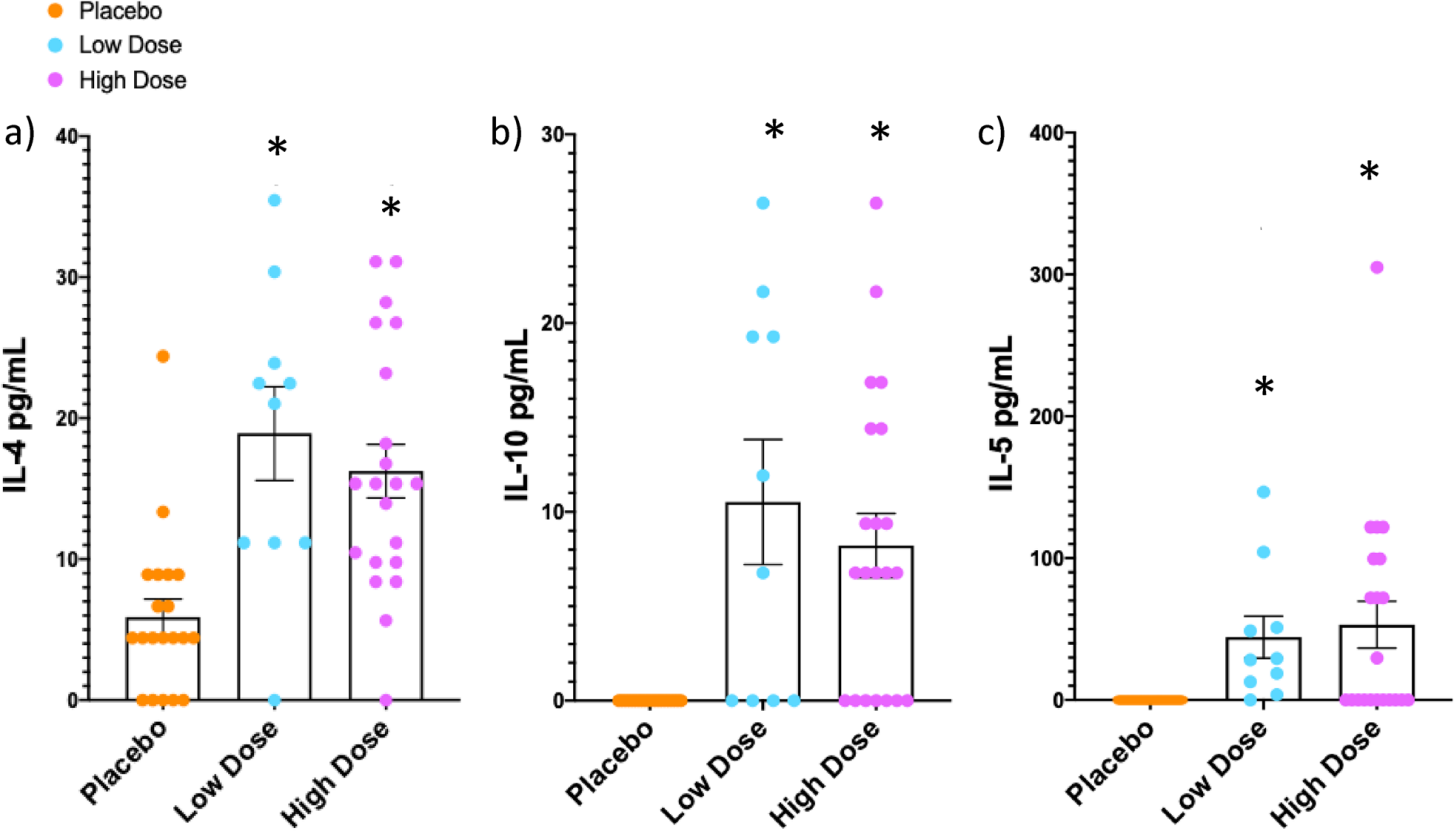
Probiotic intervention alters cytokines following social stress. Following repeated social defeat, circulating concentrations (pg/mL) of IL-4 (**a**), IL-10 (**b**), and IL-5 (**c**) were significantly increased in hamsters treated with both doses of the probiotic (low dose, *n* = 7, blue dots) (high dose, *n* = 20, purple dots) compared with placebo (*n* = 20, orange dots) (*significantly higher than placebo, *p* < 0.01). Data presented as mean +/− standard error of the mean.

A Bio-Plex Pro™ Rat Cytokine 23-Plex Assay was used to analyze circulating levels of cytokines. A rat assay was chosen because no equivalent multiplex assay exists for hamster. Only serum concentrations (pg/mL) of IL-7, IL-4, IL-10, GRO/KC, IL-5, and MIP-3α were detected in the majority of hamsters and were thus able to be reliably analyzed. Hamsters whose concentrations were out of range were given a value of 0 for analysis. It should be noted that the majority of the values were on the low end of the standard curve, which can increase variability and decrease reproducibility of results. No effect of treatment was found for IL-7, GRO/KC, and MIP-3α. One-way ANOVA revealed an effect of treatment for the anti-inflammatory cytokine IL-4 (*F*(2, 48) = 12.12, *p* < 0.0001) and Tukey’s post hoc analysis revealed that hamsters treated with both the low and high dose of the probiotic had elevated IL-4 compared to placebo-treated hamsters (high dose vs. placebo, *p* = 0.0004; low dose v placebo, *p* = 0.0004; Fig. 6a). The concentrations of IL-10 and IL-5 were not normally distributed, thus the nonparametric Kruskal–Wallis with Dunn’s multiple comparisons test was used and revealed a significant effect of treatment for IL-10 (*H*(3,51) = 19.29, *p* < 0.0001) and IL-5 (*H*(3,51) = 19.66, *p* < 0.0001). Hamsters given either dose of the probiotic had higher concentrations of both the anti-inflammatory cytokine IL-10 and the anti-inflammatory chemokine IL-5 compared to hamsters treated with placebo (Dunn’s multiple comparisons test; IL-10, high dose v placebo, *p* = 0.002, low dose v placebo, *p* = 0.0021, Fig. 6b; IL-5, high dose v placebo, *p* = 0.003, low dose v placebo, *p* = 0.0002, Fig. 6c).

## Discussion

Our results demonstrate that a probiotic intervention at a dose that is equivalent to that used in humans can induce *increases* in social avoidance, decreases in social interaction, alterations in the gut microbial community, along with modest changes in serum anti-inflammatory cytokines in hamsters. We selected the commercially available probiotic CEREBIOME® (formerly called Probio’stick®) containing the organisms *Lactobacillus helveticus* R0052 and *Bifidobacterium longum* R0175 based on previous published findings that this probiotic at similar doses can reduce stress responses following water submersion or maternal separation^37,38^ as well as anxiety- and depressive-like behavior in humans and other animals^39–42^. Thus, our behavioral findings contrast with the previous literature and were opposite of what was expected^33,43–50^. One possible explanation for our unexpected finding may be that our behavioral endpoint measures something different than do the standard tests of anxiety-like behavior such as the open field or light–dark box tests that are commonly used in mice and other rodents^45,48^. It is possible that the response to an ethologically relevant stressor, such as social stress, is very different than that observed after exposure to a more artificial stressor. It may also be the case that the effects of probiotics are specific and do not necessarily translate across strains or species. This possibility is supported by evidence that different mouse strains exhibit strain-specific probiotic effects^45,48^. Ultimately, it is unclear why our data differ from the majority of the previous literature indicating that probiotics are anxiolytic. At the very least our data serve as a caution that the behavioral effect of putative anxiolytic probiotic treatments may not always be as predicted. A careful examination of differences in probiotic-induced changes to the gut microbial community and/or cytokine signaling, however, is needed to help elucidate why probiotic intervention at varying doses drives different responses in different models.

The unweighted UniFrac metric revealed that the microbial composition of all treatment groups differed from one another prior to stress (but after 14 days of probiotic treatment). Differences in microbiota based on treatment but before behavioral manipulation suggest that probiotic intervention in the absence of stress is sufficient to alter the microbial composition. Further, in addition to the anxiogenic behavioral profile observed in hamsters treated with a low dose of the probiotic, an altered microbial profile was observed in this group compared to the high dose-treated and placebo-treated hamsters after repeated social defeat. This indicates that the social stress effects on both microbiota and behavior seem to be altered differently by the same probiotic given at different doses. This finding underscores the potential importance of dosage in the ultimate outcome of probiotic treatment.

Alpha diversity, a measure of microbial richness and abundance, was assessed using a phylogeny-based (Faith PD Whole Tree) and non-phylogeny-based (Observed OTUs) measurement. The measurements complimented one another, with both demonstrating a reduction in richness in hamsters given the low dose versus the high dose or the placebo. It is possible that a reduction in alpha diversity following social defeat in hamsters given the low dose of the probiotic drives, in part, the anxiogenic behavioral profile observed in this group^51,52^. This is an interesting possibility that should be examined further in future studies. Alternatively, it is also possible that the excipients present in a higher dose in the placebo and high dose probiotic groups drove greater microbiobial richness. This possibility is discussed further below (see paragraph on limitations).

LEfSe analysis identifies particular microbial taxa that drive differences in beta and alpha diversity across groups. Although several microbial taxa were altered between treatment groups or within groups following defeat, there were fewer changes observed compared to our previous study assessing the gut microbial community of hamsters following acute and repeated social defeat with no probiotic intervention^32^. One possibility is that the excipients (xylitol, maize-derived maltrodextrin, plum flavor, and malic acid) present in both the placebo and probiotic solutions, themselves, had some effect on the gut microbiota and that this, in turn, buffered or masked the effect of social stress on the gut microbiota. The latter possibility is supported by the recent demonstration that maltodextrin is a stressor for the gut^53^. In the present study, genus *Bifidobacterium* was significantly higher in hamsters treated with a low dose of the probiotic after the acute defeat. *Bifidobacterium longum* is present in the probiotic formulation; thus, ingestion of the probiotic at a low dose may have allowed for heightened colonization of *Bifidobacterium* in the gut and/or increased proliferation of *Bifidobacterium* species. It is not clear why this did not also occur following administration of the high dose probiotic. The phyla Proteobacteria was significantly higher in placebo-treated hamsters following repeated social stress and genus *Prevotella* was higher in hamsters given the high dose of the probiotic compared to their pre-defeat baseline. In our previous study, both Proteobacteria and *Prevotella* predicted dominance in an agonistic encounter^32^, and other research suggests these taxa may be beneficial or stress-protective for the host^50,54^. Therefore, the greater abundance of Proteobacteria or *Prevotella* in hamsters treated with the placebo or high dose probiotic may, in part, have driven the reduced behavioral response to social stress observed in these two groups.

There are some important limitations of this study that should be recognized in the context of the above discussion, however. The first, mentioned above, is that the low dose probiotic group also ingested lower doses of the excipients than did the placebo and high dose probiotic groups, which received equal concentrations of the excipients. Thus, it is possible that the lower levels of these additives in the low-dose group underlies the differential effects on the microbiome and behavior. We were not initially concerned about this because there are ample data indicating that these excipients do not have behavioral or physiological effects, including a recent finding in humans that ingestion of 75 g of sugar but *not* 75 g of maltodextrin increases the stress response to the Trier Social Stress Test compared to placebo^55^. In fact, these additives are often used as control treatments and are classified as inert by the United States Food and Drug Administration. As noted above, however, there are some recent data suggesting that maltrodextrin, at least, can alter the gut microbiome^56^. Future studies should certainly take into account the possibility that excipients might have independent effects, and it is critical for the field that this possibility be examined directly. A related limitation of this study is that the effect of treatment was only examined in socially stressed animals. Future research should determine whether probiotic or excipient treatment alters social avoidance or social investigation in unstressed individuals. Additionally, the concentrations of the probiotic bacterial strains were only examined in fecal samples and not in the gut, itself, which would provide a better measure of the actual colonization by these bacteria. Given that we used a repeated defeat stressor and collected samples before, during, and after social stress, obtaining gut samples, such as from caecum, was not possible. Finally, there is a recent report that treatment with CEREBIOME® decreases food intake and body weight gain in male but not in female rats^57^. This suggests that, going forward, measures of food intake and body mass should be included in studies such as this one.

It has been suggested that gut microbiota may drive differences in social behavior by altering cytokine signaling, a well-characterized route of communication between the gut and brain^3,29,58^. Thus, we examined whether an increase in the expression of pro-inflammatory cytokines was correlated with changes to the gut microbial community and increased behavioral susceptibility to social defeat following administration of the low dose of the probiotic. This prediction was based on previous evidence indicating that increases in proinflammatory cytokines exacerbate depressive- and anxiety-like behavior in animal models^59–65^. Unfortunately, many of the cytokines and chemokines targeted in the Luminex assay were out of detectable range in one or more groups. Thus, we were unable to analyze several of the cytokines in which we had the most interest such as IL-6 and TNFα. Of the six cytokines or chemokines that were detectable (IL-7, IL-4, IL-10, GRO/KC, IL-5, MIP-3α), three were significantly altered by treatment. Although we observed an effect of probiotic dose on behavior and on the gut microbial community, this dose-dependency was not apparent when analyzing circulating cytokine signaling. Three anti-inflammatory cytokines, IL-4, IL-10 and IL-5, were significantly elevated in both probiotic (low and high dose) groups compared to placebo. The proinflammatory cytokines IL-7 and MIP-3α and proinflammatory chemokine GRO/KC were not altered across groups. These results support previous work showing an anti-inflammatory effect of *Lactobacillus helveticus* R0052 and *Bifidobacterium longum* R0033 and R0175^66,67^. Therefore, a therapeutic benefit of probiotic treatment may be to bias the immune system toward an anti-inflammatory profile following stress. Given the finding that we observed so few changes in cytokine concentrations, however, it could be the case that the use of the rat multiplex assay to assess hamster cytokines/chemokines limited our findings, that our model of social stress is too mild, that the current probiotic is ineffective in causing a robust increase in pro- or anti-inflammatory signaling and/or that it is not via alterations in immune signaling that the probiotics altered behavior in this study.

Collectively, the results of the current study demonstrate that the effect of probiotics on behavior and on gut microbial composition are likely species- and dose-dependent. This highlights the importance of testing the effect of probiotics in multiple animal models and in various environmental and behavioral contexts. Further, the dose-dependent differences in behavior and alterations to gut microbiota following probiotic treatment clearly illustrate that a higher probiotic dose does not necessarily predict a greater behavioral response. We have demonstrated that probiotic intervention can alter both behavioral responses to social stress and gut microbiota, but future work is necessary to establish whether the changes to the gut microbiota are necessary for probiotic-induced behavioral alterations and by what mechanism these changes occur.

## Methods

### Animals

Adult male Syrian hamsters (*Mesocricetus auratus*), weighing between 120 and 130 g, were obtained from Charles River Laboratory (Kingston, NY) at approximately 3 months of age. Hamsters were group-housed until the beginning of the experiment when they were individually housed in polycarbonate cages (24 × 33 × 20 cm). Animals were housed on corncob bedding, given cotton nesting material, and maintained in a temperature-controlled colony room under a 14:10 h light/dark cycle, which is standard to maintain reproductive gonadal status in hamsters. It is important to note that individual housing is not stressful for Syrian hamsters^68^. Food and water were available ad libitum. All hamsters were handled daily for 7 days to acclimate them to handling stress before the beginning of the experiment. All protocols and procedures were approved by the Georgia State University Institutional Animal Care and Use Committee prior to experimentation, and all methods align with the National Institutes of Health Guide for the Care and Use of Laboratory Animals.

### Probiotic intervention

At the beginning of the study, group-housed Syrian hamsters (n = 50) were matched by weight within cages and randomly assigned across treatment groups from each cage and individually housed. Hamsters were then treated with either the commercial probiotic formulation CEREBIOME® (Lallemand Health Solutions Inc., Montreal, QC, Canada) containing freeze-dried lactic acid bacteria strains, *Lactobacillus helveticus* R0052 and *Bifidobacterium longum* R0175 mixed with excipients (xylitol, maize derived matrodextrin, plum flavor, and malic acid) or the placebo formulation containing the excipients. Hamsters were assigned to one of three treatment groups: placebo, probiotic at a low dose of 10^9^ colony forming units per day that is thought to be comparable to a dose normally consumed by humans, or probiotic high dose (tenfold higher than low dose) of 10^10^ colony forming units per day. Placebo and probiotic solutions were prepared prior to administration as per manufacturer instructions following careful procedures to prevent cross-contamination of treatments (for a full description, see Myles et al.^42^). Each hamster received a daily dose of 0.2 g in distilled water. Hamsters received either the probiotic intervention or placebo for 14 days prior to the behavior experiment and throughout testing for a total treatment length of 21 days. Each day, hamsters were given 0.3 mL of the appropriate solution by syringe feeding^69^, which all animals readily ingested, at the start of the active phase of the daily activity cycle.

### Behavioral procedures

All behavioral manipulations were conducted during the dark phase of the daily light:dark cycle to control for circadian variation in behavior and because this is when hamsters are active and exhibit the majority of their agonistic behavior. All hamsters were moved into the behavior suite 30 min prior to any manipulation to allow time to acclimate. Behavior trials were run under dim red light and were recorded with a CCD camera.

For acute defeat training, hamsters were placed in the home cage of a novel, same-sex aggressor for 15 min^34^. For repeated defeat training, hamsters were placed in a novel, same-sex resident aggressor’s home cage for 5 min twice a day for 4 days. The first pairing occurred at the start of the dark phase and the second occurred 4 h later. A clear plastic lid was placed over the resident’s cage during each pairing to prevent escape. The resident aggressor reliably attacked the experimental subject and the latter exhibited submissive and defensive behaviors such as upright defense, flee, and tail lift^36^.

Social behavior testing (duration 5 min) took place approximately 24 h after acute and repeated defeat training, as described previously^70^. Hamsters were placed in a novel polycarbonate cage with a novel aggressor. These aggressors were confined to a small box on one side of the polycarbonate cage, allowing the subject to see, hear, and smell the aggressor, but preventing any direct contact. Testing sessions were recorded in the same manner as the defeat training and were later analyzed by observers blinded to condition to determine the time spent “far” (in the opposite half of the polycarbonate cage from the caged resident aggressor), which we operationally define as social avoidance as described previously^70,71^, and time spent in social interaction (defined as nose to caged aggressor). Social behavior comparisons were analyzed by Repeated Measures, Two-way ANOVA with Tukey’s post hoc analysis on GraphPad Prism 8.2.0 (GraphPad Software, La Jolla, CA) and effect sizes were calculated. Differences in post hoc analyses were denoted as significant at *p < 0.05. While published reports indicate that the incidence of adverse events associated with these treatments are extremely unlikely^72^, hamsters were monitored daily throughout treatment and testing by the experimenters and the Department of Animal Resources staff, as well as veterinary technicians or veterinarians to assess coat quality and to make sure that food and water intake remained constant. Additionally, experimenters carefully observed all hamsters during each agonistic encounter for coprophagia and for any injury. No coprophagia or tissue damage occurred during training or testing.

### Fecal collection and microbiota composition analysis by 16S rRNA gene sequencing

Fresh fecal samples were collected just before the beginning of the active (dark) phase of the daily light:dark cycle at three time points: 1) prior to the initial defeat (baseline samples), 2) 24 h after the acute defeat (acute defeat samples), and 3) 24 h after the final defeat (repeated defeat sample) to assess the microbial community before any stress, after one bout of social defeat, and after repeated bouts of social defeat. To avoid additional stress to the animal, hamsters were transferred into a clean cage and fecal samples were collected from the bedding approximately 1 h later. Samples were collected in RNase-free microcentrifuge tubes and were immediately frozen and stored at − 80 °C until further processing.

Characterization of microbial communities was performed by 16S rRNA gene sequencing as previously described^32,73^. Briefly, extracted DNA was used to construct sequencing libraries according to Illumina’s “16 S Metagenomic Sequencing Library Preparation” guide (Part # 15044223 Rev. B), with the exception of using Qiagen HotStar MasterMix for the first PCR (“amplicon PCR”) and halving reagent volumes for the second PCR (“index PCR”). The template specific primers were (without the overhang adapter sequence) the following: forward (5′-CCTACGGGNGGCWGCAG-3′) and reverse (5′-GACTACHVGGGTATCTAATCC-3′), targeting the V3–V4 hypervariable region^74^ specific to bacterial organisms and generating a fragment of around 460 bp. The first PCR (“amplicon PCR”) was carried out for 25 cycles with annealing temperatures of 55 °C. Diluted pooled samples were loaded on an Illumina MiSeq and sequenced using a 500-cycle (paired-end sequencing configuration of 2 × 250 bp) MiSeq Reagent Kit v3.

### 16S rRNA gene sequencing analysis

The sequences were demultiplexed and quality filtered using the Quantitative Insights Into Microbial Ecology 2 (QIIME 2, version 3.5.5) software package^75^. Forward and reverse Illumina reads were joined using the fastq-join method. We used the QIIME 2 default parameters for quality filtering^75^. Sequences were clustered using amplicon sequencing variant tables with Deblur^76^. Clusters were then classified taxonomically using the Greengenes reference database, Version 13.5^77^. Clusters that did not match any Greengenes Operational taxonomic units (OTUs) were kept. A single representative sequence for each OTU was aligned and a phylogenetic tree was built using FastTree^78^.

The phylogenetic tree described above was used to assess beta and alpha diversity^79,80^. Unweighted UniFrac^81^ was used to compute distances and to measure beta diversity between groups using rarefied OTU table count. Principal coordinates analysis (PCoA) plots were used to further assess and visualize beta diversity. Groups were compared for distinct clustering using PERMANOVA method using vegan R-package through QIIME 2. The phylogeny-based metric, phylogenetic diversity whole tree (PD whole tree) measurement and the non phylogeny-based metric, Observed OTUs, were determined with QIIME 2. Kruskal–Wallis with Dunn’s multiple comparison test was used to determine differences among groups on GraphPad Prism 8.2.0 (GraphPad Software, La Jolla, CA). Lastly, LEfSE (Linear Discriminate Analysis Effect Size) was used to compare abundance of specific taxa between groups^82^.

### Analysis of fecal samples for probiotic strains via quantitative PCR

The concentrations of the specific bacterial strains fed to the hamsters was measured from fecal samples using qPCR methodology and primers as described in detail in Myles et al.^42^.

### Multiplex assay procedure

24 h after the final defeat and immediately following behavior testing, hamsters were briefly anesthetized with isoflurane and euthanized by cervical dislocation. Trunk blood was collected and allowed to clot at room temperature for 2 h. After 2 h, blood was centrifuged for 20 min at 2000×*g* to obtain serum, which was immediately frozen and stored at − 80 °C.

A Bio-Plex Pro™ Rat Cytokine 23-Plex Assay was conducted on hamster serum using a fluorescent bead-based instrument Bio-Plex 200 (Bio-Rad, Hercules, CA). Bio-Plex instruments were validated using a Bio-Plex Validation kit 24 h prior to conducting the assay, and instruments were calibrated immediately prior to performing the assay using a Bio-Plex Calibration Kit. The assay was conducted per the manufacturer’s protocol using the recommended sample dilution (4×) and standard curve concentrations. All samples and standards were assayed in duplicates. This multiplex technology uses “xMAP”-based microspheres to detect protein concentrations of target analytes. The concentration of each analyte was determined by Bio-Plex Manager, and the analyte concentrations were compared across groups by ANOVA with Tukey’s post hoc comparisons or Kruskal–Wallis with Dunn’s multiple comparisons test on GraphPad Prism 8.2.0 (GraphPad Software, La Jolla, CA). A rat assay was chosen because no equivalent multiplex assay exists for hamster. Notably, most of the sequences for the analytes in the multi-plex assay share a 77–90% homology with hamster sequences, suggesting a high likelihood of cross-reactivity with hamster proteins.

## Data Availability

All raw sequence data resulting from the 16S rRNA gene sequencing are available on NCBI under accession number PRJNA669011.
